# Immunhistochemische Analyse einer Hypoxie-assoziierten Signatur in Melanomen mit positivem und negativem Schildwächterlymphknoten: Hypoxie-assoziierte Signatur primär kutaner Melanome

**DOI:** 10.1007/s00105-021-04934-x

**Published:** 2022-01-07

**Authors:** Ferdinand Toberer, Julia K. Winkler, Holger A. Haenssle, Monika Heinzel-Gutenbrunner, Alexander Enk, Wolfgang Hartschuh, Peter Helmbold, Heinz Kutzner

**Affiliations:** 1 Universitäts-Hautklinik Heidelberg, Ruprecht-Karls-Universität Heidelberg, Im Neuenheimer Feld 440, 69120 Heidelberg, Deutschland; 2 MH-Statistical Consulting, Marburg, Deutschland; 3 Dermatopathologie Bodensee, Friedrichshafen, Deutschland

**Keywords:** Malignes Melanom, Schildwächterlymphknoten, Immunhistochemie, Metabolische Signatur, HIF-1α, Malignant melanoma, Sentinel node, Immunohistochemistry, Metabolic signature, HIF-1alpha

## Abstract

Metabolische Anpassungsprozesse, vermittelt durch sog. Hypoxie-induzierbare Faktoren und deren Zielgene, spielen in zahlreichen Malignomen eine wichtige Rolle. Rasch wachsende Tumoren können ihre Stoffwechselvorgänge an eine auftretende Hypoxie anpassen. So werden beispielsweise nach der Aktivierung des „Hypoxia inducible-factors-1α“ Modifikationen am Glukosestoffwechsel, der intrazellulären pH-Regulation oder der Angiogenese initiiert. In dieser immunhistochemischen Pilotstudie analysierten wir primär kutane Melanome mit positivem und negativem Schildwächterlymphknotenstatus im Hinblick auf mögliche Unterschiede ihrer metabolischen Signatur. Hierbei konnten wir unter anderem zeigen, dass die Expression von Glukosetransporter‑1 (GLUT-1) sowohl in allen Melanomen ohne Subgruppenanalyse, als auch in der Subgruppe mit negativem Schildwächterlymphknoten positiv mit der Tumordicke sowie dem Vorliegen einer Ulzeration korrelierte. Zudem korrelierte bei Melanomen mit positivem Schildwächterlymphknoten die Expression von vaskulärem endothelialem Wachstumsfaktor (VEGF) positiv mit dem Vorliegen einer Ulzeration.

## Abstract (en)

Metabolic reprogramming mediated by hypoxia-inducible factors and its downstream targets plays a crucial role in many human malignancies. Excessive proliferation of tumor cells under hypoxic conditions leads to metabolic reprogramming and altered gene expression enabling tumors to adapt to their hypoxic environment. Here we analyzed the metabolic signatures of primary cutaneous melanomas with positive and negative sentinel node status in order to evaluate potential differences in their metabolic signature. We found a positive correlation of the expression of glucose transporter 1 (GLUT-1) with tumor thickness and ulceration in all melanomas with subgroup analyses as well as in the subgroup with a negative sentinel node. Furthermore, the expression of vascular endothelial growth factor (VEGF) was positively correlated with the presence of ulceration in melanomas with positive sentinel node.

Obwohl primär kutane Melanome nur 5 % aller maligner Hauttumoren repräsentieren, sind sie für mehr als zwei Drittel aller mit Hauttumoren assoziierten Todesfälle verantwortlich [1]. Hypoxie als Folge einer exzessiven Tumorzellproliferation, die entfernt von einem gut entwickelten Gefäßsystem stattfindet, spielt eine zentrale Rolle in der Karzinogenese [2]. Der sog. „hypoxia inducible factor-1α“ (HIF-1α) orchestriert die Transkription von über 200 Zielgenen, die für das Tumorwachstum unter hypoxischen Bedingungen verantwortlich sind [2, 3]. Die nachfolgend aktivierten Signalwege induzieren verschiedenste Prozesse wie beispielsweise Angiogenese, eine veränderte Energiegewinnung (Glykolyse statt oxidativer Phosphorylierung) oder Zellproliferation [2, 4, 5].

Der vaskuläre endotheliale Wachstumsfaktor (VEGF) stellt ein zentrales Zielgen von HIF-1α dar, vermittelt die physiologische und pathologische Angiogenese und ist auch therapeutisch als potenzielle Zielstruktur von Interesse [5, 6].

Der Glukosetransporter‑1 (GLUT-1) ist der häufigste humane Glukosetransporter und wird in rasch proliferierenden Tumorzellen vermehrt exprimiert, um einen gesteigerten Glukosebedarf decken zu können [2, 7].

Eine verstärkte Expression von Monocarboxylattransportern (MCTs) wurde in zahlreichen malignen Tumoren beschrieben. Da sie unter anderem die Aufnahme von Chemotherapeutika in Tumorzellen steuern, könnte ihre Expression auch mit dem Therapieansprechen korrelieren [8].

Die carbonische Anhydrase IX (CAIX) ist ein HIF-1α-induzierter pH-Regulator, der zur geänderten Stoffwechsellage von Tumorzellen beiträgt und mit Metastasierung und schlechter Prognose bei zahlreichen malignen Tumoren, unter anderem dem malignen Melanom, assoziiert ist [9–11].

Durch die zunehmende Verfügbarkeit adjuvanter Therapieansätze beim malignen Melanom mit lymphatischer Metastasierung wandelt sich die Bedeutung der Schildwächterlymphknotenbiopsie (SNB) von einem Prognosemarker zu einer Stagingmaßnahme, die weitere adjuvante Therapieoptionen definiert [12, 13]. Dennoch bleibt die Auswahl der Patienten, die eine SNB erhalten sollen, eine Herausforderung, da etwa 80 % aller Melanompatienten, die eine solche Operation erhalten, einen negativen Befund aufweisen [13].

Die vorliegende immunhistochemische Pilotstudie vergleicht erstmals systematisch die metabolische Signatur von primär kutanen Melanomen mit positivem und negativem Schildwächterlymphknoten. Hierfür wurde die Expression von HIF-1α und einiger zentraler Zielgene (VEGF, MCT4, GLUT‑1 und CAIX) untersucht. Diese Studie repräsentiert einen zusätzlichen Schritt zur Analyse der metabolischen Signatur des malignen Melanoms und soll helfen, weitere Studien zu initiieren, deren Ziel die Definition von metabolischen Biomarkern ist, die den Sentinelstatus bei primär kutanen Melanomen vorherzusagen helfen könnten.

## Material und Methoden

### Gewebeproben

Insgesamt wurden 34 formalinfixierte und in Paraffin eingebettete primär kutane Melanome immunhistochemisch untersucht, wobei 15 einen positiven und 19 einen negativen Schildwächterlymphknoten aufwiesen. Sämtliche Tumoren entstammen dem dermatopathologischen Archiv der Universitätshautklinik Heidelberg aus dem Zeitraum 2015 bis 2019. Es wurde darauf geachtet, dass sowohl die klinischen (Alter, Geschlecht) als auch die histologischen (Tumordicke, Tumorsubtyp, Ulzeration) Merkmale der Primärtumoren in beiden Gruppen gleichmäßig verteilt waren. Die Studie wurde von der Ethikkommission der Universität Heidelberg genehmigt (S-091/2011) und in Übereinstimmung mit der Helsinki Deklaration von 1975 (aktualisiert 2000) durchgeführt. Die Tab. 1 gibt einen Überblick über die klinischen und histologischen Merkmale der analysierten Tumoren.

**Table 1:** 

Merkmal		SNS^+^ (*n* = 15)	SNS^−^ (*n* = 19)
*Alter*	Median; Mittelwert ± SD	60; 60,5 ± 16,38 Jahre	66; 66,1 ± 11,09 Jahre
*Geschlecht*	Weiblich vs. männlich	9:6	11:8
*pT*	T1a/T1b/T2a/T2b/T3a/T3b/T4a/T4b	0/0/3/0/0/3/2/7	0/0/3/0/3/1/2/10
*Histotyp*	SSM//NM/ALM	4/10/1	2/16/1
*Tumordicke*	Median; Mittelwert ± SD	4,5; 5,1 ± 2,8 mm	4,8; 4 ± 2,04 mm
*Ulzeration*	Ja vs. nein	10:5	11:8
*Lokalisation*	Rumpf/Arm/Bein/Kopf/Fuß/plantar	9/0/3/2/0/1	10/2/2/1/2/2

*SNS*^*+*^ positiver Schildwächterlymphknoten, *SNS*^*−*^ negativer Schildwächterlymphknoten, *SD* Standardabweichung, *SSM* superfiziell spreitendes Melanom, *NM* noduläres Melanom, *ALM* akral-lentiginöses Melanom, *pT* pathologisches Stadium Primärtumor

## Immunhistochemie

Die Tab. 2 zeigt im Detail die verwendeten Antikörper, ihre Verdünnung und die entsprechende Vorbehandlung. Zur Bestimmung der Proteinexpression wurde ein bereits etablierter, semiquantitativer, immunhistochemischer Quantifizierungsscore verwendet, der sich aus dem Produkt der prozentualen Anzahl positiv gefärbter Tumorzellen (prozentualer Anteil positiver Zellen: 0: 0 %, 1: bis 1 %, 2: 2–10 %, 3: 11–50 % und 4: > 50 %) und der Färbeintensität (0: negativ, 1: schwach, 2: mittelstark und 3: stark) zusammensetzt [14]. Zudem wurde die epidermale Expression von VEGF anhand der Färbeintensität der Keratinozyten des Deckepithels bestimmt (0: negativ, 1: schwach, 2: mittelstark und 3: stark). Die Auszählung erfolgte lichtmikroskopisch und verblindet durch 2 unabhängige Dermatopathologen (FT und WH) ohne Kenntnis des Schildwächterstatus oder jedweder anderer klinischer Daten. Alle Fälle, in denen divergierende Quantifizierungsscores ermittelt wurden, wurden anschließend gemeinsam besprochen, um einen einheitlichen Score festzulegen.

**Table 2:** 

Antikörper	Klon	Quelle	Hersteller	Verdünnung	Vorbehandlung
VEGF	EP1176Y	Kaninchen	Zytomed Systems, Berlin	1:200	pH 9,0
HIF-1α	Polyklonal	Kaninchen	Bio-Techne, Minneapolis, USA	1:50	pH 9,0
GLUT‑1	SPM498	Maus	Zytomed Systems, Berlin	1:400	pH 6,1
MCT4	D‑1	Maus	Santa Cruz Biotechnology, Heidelberg	1:100	pH 9,0
CAIX	Polyklonal	Kaninchen	Abcam, Cambridge, USA	1:200	pH 6,1

## Statistik

Die statistischen Analysen wurden mit der SPSS statistical package Software (v24.0, SPSS Inc., Chicago, IL, USA) durchgeführt. Unterschiede hinsichtlich der Proteinexpression zwischen den Gruppen wurden mittels des Mann-Whitney-U-Testes mit exakten *p*-Werten ermittelt. Um die Verteilung der Proteinexpression zwischen den Schildwächterlymphknoten-positiven und -negativen Fällen zu demonstrieren, wurden Box-Plots mit Median, Interquartilsabstand und Spannweite erstellt. Um die Korrelation der Proteinexpression mit klinischen (Alter, Geschlecht) und histologischen (Tumordicke, Histotyp, Ulzeration) Merkmalen zu untersuchen, wurde der Korrelationskoeffizient nach Spearman berechnet. Dichotome Variablen (Geschlecht, Ulzeration und Sentinelstatus) wurden zusätzlich mit Kreuztabellen und dem Exakt-Test nach Fisher analysiert. *p*-Werte < 0,05 wurden als statistisch signifikant gewertet.

## Ergebnisse

### Klinische und histologische Daten

Die klinischen und histologischen Daten sind in Tab. 1 zusammengefasst. Das mittlere Alter unterschied sich nicht zwischen den Patienten mit positivem und negativem Schildwächterlymphknoten (*p* = 0,246). Die Tumordicke (Mittelwert ± SD) bei Tumoren mit positivem Schildwächterlymphknoten (5,1 ± 2,7) und solchen mit negativem Befund (4,4 ± 2,0) unterschied sich ebenfalls nicht signifikant (*p* = 0,463). Es zeigte sich keine Korrelation von Geschlecht, Histotyp und Ulzeration mit dem Status des Schildwächterlymphknotens (*p* = 1, 0,573 und 1), was für eine gleichmäßige Verteilung dieser Merkmale in beiden Gruppen spricht.

## HIF-1α-Expression

**Figure 1:**
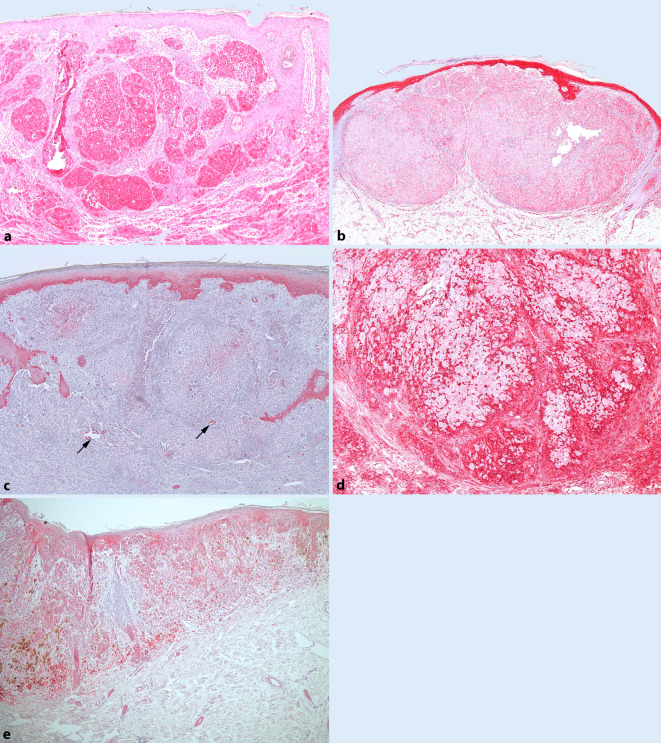

HIF-1α wurde in allen untersuchten Tumoren exprimiert. Der QS (Mittelwert ± SD) unterschied sich nicht signifikant zwischen Tumoren mit positivem (8,86 ± 3,56) und Tumoren mit negativem Schildwächterbefund (8,94 ± 3,04; *p* = 0,944) (Abb. 1a und 2a).

**Figure 2:**
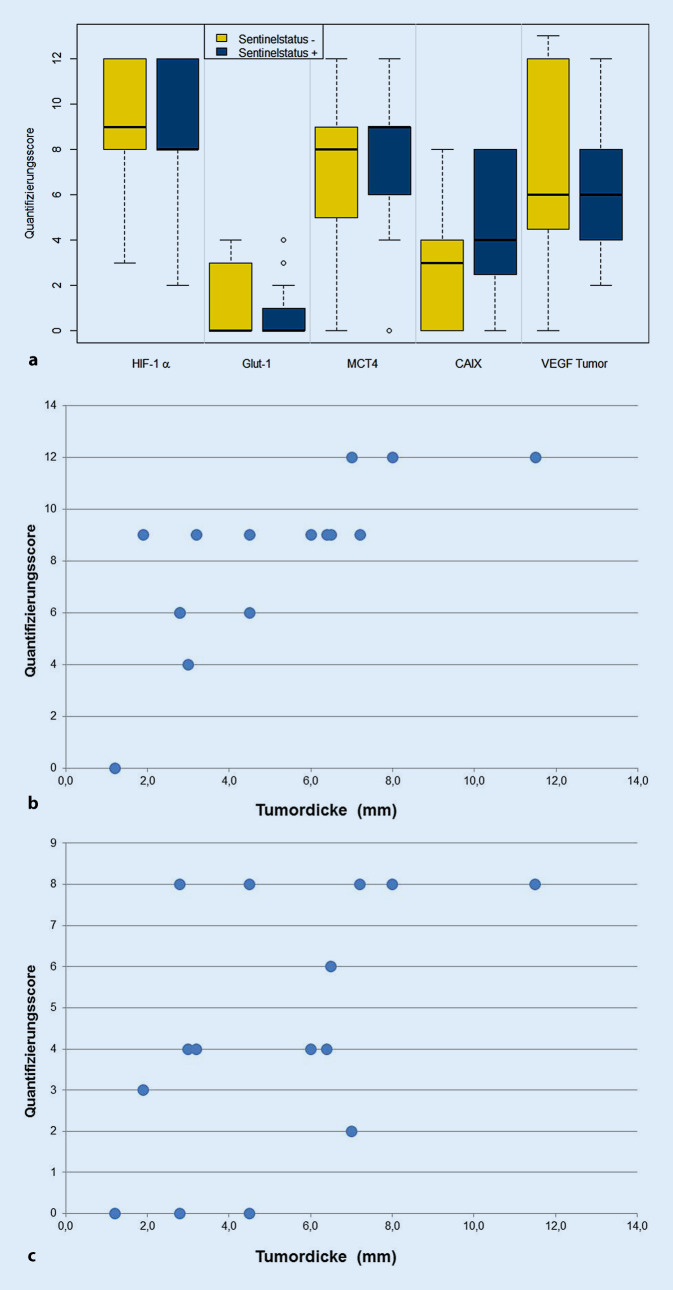

## VEGF-Expression

Der QS der tumoralen VEGF-Expression in Melanomen mit positiven Schildwächterlymphknoten (5,93 ± 2,65) unterschied sich nicht signifikant vom QS in Schildwächterlymphknoten-negativen Tumoren (7,10 ± 4,29; *p* = 0,337). Eine epidermale VEGF-Expression fand sich in allen Tumoren, wobei 91 % der analysierten Melanome eine starke epidermale Expression von VEGF aufwiesen. Die epidermale VEGF-Expression unterschied sich jedoch nicht signifikant zwischen Tumoren mit positivem und negativem Schildwächterlymphknoten (*p* = 0,076). Interessanterweise wies die Epidermis direkt oberhalb der Melanome eine tendenziell stärkere VEGF-Expression auf als die Epidermis in der Peripherie (Abb. 1b und 2a).

## GLUT-1-Expression

GLUT‑1 war in 27 % (4/15) der Melanome mit positivem Wächterlymphknoten und in 32 % (6/19) der Tumoren mit negativem Sentinel exprimiert. Die Expressionslevel von GLUT‑1 zeigten hierbei keine statistisch signifikanten Unterschiede zwischen Sentinel-positiven (0,86 ± 1,55) und Sentinel-negativen Tumoren (1,15 ± 1,77), (*p* = 0,679) (Abb. 1c und 2a).

## MCT4-Expression

Die MCT4-Expression unterschied sich nicht statistisch signifikant zwischen Tumoren mit positivem Schildwächterlymphknoten (8,06 ± 3,23) und solchen mit negativem Befund (7,00 ± 3,59; *p* = 0,376). Eine Expression von MCT4 fand sich hierbei in 14/15 Schildwächterlymphknoten-positiven Tumoren (93 %) und in 17/19 Melanomen ohne Schildwächterbefall (89 %) (Abb. 1d und 2a).

## CAIX-Expression

Die CAIX-Expression zeigte keinen statistisch signifikanten Unterschied zwischen Tumoren mit Befall des Schildwächterlymphknotens (4,46 ± 3,09) und solchen mit negativem Sentinel (2,78 ± 2,76; *p* = 0,105) (Abb. 1e und 2a).

## Statistische Korrelationsanalysen

### Proteinexpression

Um mögliche Korrelationen zwischen den Expressionsscores der einzelnen Proteine zu untersuchen, wurden Korrelationskoeffizienten nach Spearman berechnet. Hierbei zeigten sich weder signifikante Korrelationen, wenn man alle Melanome zusammen betrachtete, noch wenn man die Subgruppen getrennt nach dem Status des Schildwächterlymphknotens verglich.

## Proteinexpression und klinische/histologische Daten

Keines der analysierten Proteine zeigte eine statistisch signifikante Assoziation mit dem Alter und dem Geschlecht der Patienten oder dem Histotyp. Die Expression von GLUT‑1 war ohne Subgruppenanalyse positiv mit der Tumordicke (Korrelationskoeffizient: 0,350, *p* = 0,042) sowie dem Vorliegen einer Ulzeration (Korrelationskoeffizient: 0,470, *p* = 0,005) korreliert. Auch in der Subgruppe mit negativem Schildwächterlymphknoten zeigte die GLUT-1-Expression eine positive Korrelation mit der Tumordicke (Korrelationskoeffizient: 0,535, *p* = 0,018) und dem Ulzerationsstatus (Korrelationskoeffizient: 0,511, *p* = 0,025). In der Gruppe der Melanome mit Schildwächterbefall waren die MCT4-Expression (Korrelationskoeffizient: 0,796, *p* = 0,000) und die CAIX-Expression (Korrelationskoeffizient: 0,539, *p* = 0,038) positiv mit der Tumordicke korreliert (Abb. 2b, c). Zudem war bei den Schildwächterlymphknoten-positiven Tumoren die VEGF-Expression positiv mit dem Vorliegen einer Ulzeration korreliert (Korrelationskoeffizient: 0,566, *p* = 0,028).

## Diskussion

In einer Vielzahl maligner Tumoren sind sog. Hypoxie-induzierbare Faktoren für die Umstrukturierung von Stoffwechselvorgängen (im englischen Sprachraum als „metabolic reprogramming“ bezeichnet) und die damit einhergehenden veränderten Genexpressionsmuster verantwortlich. In der vorliegenden immunhistochemischen Pilotstudie analysierten wir die Expression von HIF-1α und mehrerer seiner zentralen Zielproteine (VEGF, GLUT‑1, MCT4 und CAIX) in primär kutanen Melanomen mit positivem und negativem Schildwächterlymphknoten, um Erkenntnisse über die metabolische Signatur dieser Tumoren und deren möglichen Einfluss auf den Schildwächterstatus zu gewinnen.

Hierbei zeigte sich in allen untersuchten Melanomen eine Expression von HIF-1α, was dafür spricht, dass metabolische Anpassungsprozesse im Rahmen einer Tumorhypoxie beim malignen Melanom eine pathogenetische Rolle spielen könnten. Unsere Ergebnisse stehen im Einklang mit den Analysen anderer Arbeitsgruppen, die die HIF-1α-Expression in melanozytären und nichtmelanozytären Hauttumoren untersuchten [2, 15]. Die Inhibition von HIF-1α repräsentiert einen vielversprechenden onkologischen Therapieansatz, wobei verschiedene Methoden zur Reduktion der HIF-1α-Aktivität beschrieben wurden. Hierzu zählt insbesondere die Reduktion der HIF-1α-mRNA-Level, der DNA-Bindungskapazität oder Transkriptionsaktivität [5, 16, 17].

VEGF-induzierte Signalwege sind essenziell für die physiologische und pathologische Neubildung von Blut- und Lymphgefäßen und stellen zudem ein potenzielles therapeutisches Ziel dar [6]. Wir konnten in 32/34 Melanomen (94 %) eine VEGF-Expression durch die Tumorzellen und in allen untersuchten Tumoren eine VEGF-Expression in der überliegenden Epidermis feststellen, was auf eine mögliche pathogenetische Rolle von VEGF hindeutet. Wie kürzlich von unserer Arbeitsgruppe beim Talgdrüsenkarzinom beschrieben [17], fanden wir auch hier die höchsten epidermalen VEGF-Level im Epithel direkt oberhalb der Melanome. Dieses Phänomen reflektiert möglicherweise eine Stimulation der epidermalen VEGF-Produktion durch tumoral sezernierte Zytokine wie beispielsweise „transforming growth factor-α“ [18]. Interessanterweise fanden wir bei Melanomen mit positivem Schildwächterlymphknoten eine positive Korrelation zwischen der tumoralen VEGF-Expression und dem Vorliegen einer Ulzeration. Womöglich produzieren rasch wachsende Melanome, die klinisch zur Ulzeration neigen, besonders hohe Mengen an VEGF, um die Angiogenese zu fördern, was auch zur lymphatischen Metastasierung in den Wächterlymphknoten beitragen könnte.

Mehrere Studien konnten eine Assoziation der GLUT-1-Expression mit der Tumordicke, einer Ulzeration und reduzierten Überlebensraten beim malignen Melanom zeigen [19, 20]. Wir fanden eine GLUT-1-Expression in 27 % der Melanome mit positivem Sentinel und in 32 % der Tumoren mit negativem Sentinel. Interessanterweise war im vorliegenden Untersuchungsgut die GLUT-1-Expression positiv mit der Tumordicke und einer Ulzeration korreliert, wenn man alle Tumoren ohne Berücksichtigung des Schildwächterlymphknotens betrachtete, und das Gleiche galt für die Melanome mit negativem Wächterlymphknoten. Diese Daten könnten hinweisend dafür sein, dass die Expression von GLUT‑1 insbesondere in dickeren und/oder ulzerierten Tumoren eine Rolle im Rahmen von veränderten Stoffwechselvorgängen spielt. Nichtsdestotrotz sind weitere Studien notwendig, die sich mit der Expression von GLUT‑1 und seiner Isoformen (z. B. GLUT-3) in Melanomen befassen, da GLUT-Isoformen sowohl alternativ als auch simultan exprimiert werden können [19, 21].

Monocarboxylattransporter wie MCT4 verhindern eine toxische Laktatanreicherung innerhalb von neoplastischen Zellen, indem sie den Efflux von Laktat zusammen mit Protonen fördern [9]. Die Expression von MCT4 ist in Melanomen mit der Tumordicke, dem Mitoseindex, einem nodulären Histotyp und einem geringeren Gesamtüberleben assoziiert [9]. Dies spricht für eine wichtige pathogenetische Rolle von MCT4 beim malignen Melanom. Wir konnten eine Expression von MCT4 in 31/34 der analysierten Tumoren (91 %) finden, wobei die MCT4-Expression nicht mit einer Ulzeration oder dem Histotyp korreliert war. In Melanomen mit positivem Schildwächterlymphknoten jedoch war die MCT4-Expression positiv mit der Tumordicke korreliert, was in Übereinstimmung mit Daten von Pinheiro et al. steht, die eine ebensolche Korrelation beschrieben haben [9].

CAIX fungiert unter hypoxischen Bedingungen als ein pH-Regulator, und eine gesteigerte Expression dieses Proteins findet sich in einer Reihe von Malignomen unter anderem beim Brustkrebs, bei dem Nierenzellkarzinom, Adenokarzinomen von Zervix und Kolon, dem Bronchialkarzinom und dem malignen Melanom, wobei die CAIX-Expression mit einer schlechteren Prognose assoziiert ist [10, 11]. In der vorliegenden Studie zeigte sich eine CAIX-Expression in 25/34 Tumoren (74 %), wobei diese positiv mit der Tumordicke von Melanomen mit positivem Wächterlymphknoten korreliert war. Die Korrelation der CAIX- und MCT4-Expression mit der Tumordicke bei Melanomen mit positivem Sentinel, nicht jedoch in Tumoren mit negativem Wächterlymphknoten, könnte auf eine unterschiedliche metabolische Signatur der Melanome in Abhängigkeit vom Schildwächterlymphknotenstatus hindeuten. Eine mögliche Hypothese ist, dass dickere Melanome mit positivem Sentinel einen erhöhten Glukosemetabolismus aufweisen, was zu einem abfallenden intrazellulären pH-Wert führt, welcher wiederum eine gesteigerte Expression von MCT4 und CAIX nach sich zieht.

Die vorliegende Studie weist durchaus Limitation auf (beispielsweise retrospektive Datenanalyse, vergleichsweise geringe Fallzahl und fehlende Follow-up-Informationen), weshalb die Resultate mit Vorsicht interpretiert werden müssen. Nichtsdestotrotz erlauben unsere Daten den Schluss, dass metabolische Anpassungsprozesse bei der Pathogenese des primär kutanen Melanoms eine Rolle zu spielen scheinen. Weiterhin deuten die Ergebnisse darauf hin, dass zwischen Tumoren mit positivem und negativem Schildwächterlymphknoten Unterschiede in der Expression relevanter metabolischer Proteine wie VEGF, GLUT‑1, MCT4 und CAIX existieren, wobei diese Daten in prospektiven Studien, die eine höhere Fallzahl aufweisen und auch Follow-up-Informationen einschließen, bestätigt werden müssen. Dabei wäre es wünschenswert, dass diese Arbeiten auch die prognostische Bedeutung der Expression von HIF-1α sowie seiner Zielproteine untersuchten und einen Beitrag zur Verbesserung der präoperativen Selektion von Melanompatienten für eine Schildwächterlymphknotenbiopsie leisten könnten.

## Fazit für die Praxis

Metabolische Anpassungsprozesse, vermittelt durch sog. Hypoxie-induzierbare Faktoren und deren Zielgene, spielen in zahlreichen Malignomen eine wichtige Rolle. Rasch wachsende Tumoren können ihre Stoffwechselvorgänge an eine auftretende Hypoxie anpassen. So werden beispielsweise nach der Aktivierung des „Hypoxia inducible-factors-1α“ Modifikationen am Glukosestoffwechsel, der intrazellulären pH-Regulation oder der Angiogenese initiiert. In dieser immunhistochemischen Pilotstudie analysierten wir primär kutane Melanome mit positivem und negativem Schildwächterlymphknotenstatus im Hinblick auf mögliche Unterschiede ihrer metabolischen Signatur. Hierbei konnten wir unter anderem zeigen, dass die Expression von Glukosetransporter‑1 (GLUT-1) sowohl in allen Melanomen ohne Subgruppenanalyse, als auch in der Subgruppe mit negativem Schildwächterlymphknoten positiv mit der Tumordicke sowie dem Vorliegen einer Ulzeration korrelierte. Zudem korrelierte bei Melanomen mit positivem Schildwächterlymphknoten die Expression von vaskulärem endothelialem Wachstumsfaktor (VEGF) positiv mit dem Vorliegen einer Ulzeration.

## References

[1] Siegel RL, Miller KD, Jemal A (2017). Cancer statistics, 2017. CA Cancer J Clin.

[2] Seleit I, Bakry OA, Al-Sharaky DR, Ragab RAA, Al-Shiemy SA (2017). Evaluation of hypoxia inducible factor-1α and glucose transporter-1 expression in non melanoma skin cancer: an immunohistochemical study. J Clin Diagn Res.

[3] Shay JE, Celeste Simon M (2012). Hypoxia-inducible factors: crosstalk between inflammation and metabolism. Semin Cell Dev Biol.

[4] Loor G, Schumacker PT (2008). Role of hypoxia-inducible factor in cell survival during myocardial ischemia-reperfusion. Cell Death Differ.

[5] Dondajewska E, Suchorska W (2011). Hypoxia-inducible factor as a transcriptional factor regulating gene expression in cancer cells. Współczesna onkologia.

[6] Smith NR, Baker D, James NH, Ratcliffe K, Jenkins M, Ashton SE (2010). Vascular endothelial growth factor receptors VEGFR-2 and VEGFR-3 are localized primarily to the vasculature in human primary solid cancers. Clin Cancer Res.

[7] Gatenby RA, Smallbone K, Maini PK, Rose F, Averill J, Nagle RB (2007). Cellular adaptations to hypoxia and acidosis during somatic evolution of breast cancer. Br J Cancer.

[8] Baltazar F, Pinheiro C, Morais-Santos F, Azevedo-Silva J, Queiros O, Preto A (2014). Monocarboxylate transporters as targets and mediators in cancer therapy response. Histol Histopathol.

[9] Pinheiro C, Miranda-Gonçalves V, Longatto-Filho A, Vicente AL, Berardinelli GN, Scapulatempo-Neto C (2016). The metabolic microenvironment of melanomas: prognostic value of MCT1 and MCT4. Cell Cycle.

[10] Shin HJ, Rho SB, Jung DC, Han IO, Oh ES, Kim JY (2011). Carbonic anhydrase IX (CA9) modulates tumor-associated cell migration and invasion. J Cell Sci.

[11] Chiche J, Ilc K, Brahimi-Horn MC, Pouyssegur J (2010). Membrane-bound carbonic anhydrases are key pH regulators controlling tumor growth and cell migration. Adv Enzyme Regul.

[12] Peach H, Board R, Cook M, Corrie P, Ellis S, Geh J (2020). Current role of sentinel lymph node biopsy in the management of cutaneous melanoma: a UK consensus statement. J Plast Reconstr Aesthet Surg.

[13] Manninen AA, Gardberg M, Juteau S (2019). BRAF immunohistochemistry predicts sentinel lymph node involvement in intermediate thickness melanomas. PLoS ONE.

[14] Brost S, Koschny R, Sykora J, Stremmel W, Lasitschka F, Walczak H (2010). Differential expression of the TRAIL/TRAIL-receptor system in patients with inflammatory bowel disease. Pathol Res Pract.

[15] Marconi A, Borroni RG, Truzzi F, Longo C, Pistoni F, Pellacani G (2015). Hypoxia-inducible factor-1α and CD271 inversely correlate with melanoma invasiveness. Exp Dermatol.

[16] Brocato J, Chervona Y, Costa M (2014). Molecular responses to hypoxia-inducible factor 1α and beyond. Mol Pharmacol.

[17] Toberer F, Haenssle HA, Rütten A, Kazakov D, Kastnerova L, Enk A (2019). Angiogenesis in ocular and extraocular sebaceous carcinoma. Acta Derm Venereol.

[18] Johnson KE, Wilgus TA (2012). Multiple roles for VEGF in non-melanoma skin cancer: angiogenesis and beyond. J Skin Cancer.

[19] Ruby KN, Liu CL, Li Z, Felty CC, Wells WA, Yan S (2019). Diagnostic and prognostic value of glucose transporters in melanocytic lesions. Melanoma Res.

[20] Yan S, Coffing BN, Li Z, Xie H, Brennick JB, Beg HA (2016). Diagnostic and prognostic value of proex C and GLUT1 in melanocytic lesions. Anticancer Res.

[21] Parente P, Coli A, Massi G, Mangoni A, Fabrizi MM, Bigotti G (2008). Immunohistochemical expression of the glucose transporters GLUT-1 and GLUT-3 in human malignant melanomas and benign melanocytic lesions. J Exp Clin Cancer Res.

